# Echinoderms Display Morphological and Behavioural Phenotypic Plasticity in Response to Their Trophic Environment

**DOI:** 10.1371/journal.pone.0041243

**Published:** 2012-08-01

**Authors:** Adam D. Hughes, Lars Brunner, Elizabeth J. Cook, Maeve S. Kelly, Ben Wilson

**Affiliations:** Department of Ecology, Scottish Association for Marine Science, Oban, Argyll, Scotland; University of Otago, New Zealand

## Abstract

The trophic interactions of sea urchins are known to be the agents of phase shifts in benthic marine habitats such as tropical and temperate reefs. In temperate reefs, the grazing activity of sea urchins has been responsible for the destruction of kelp forests and the formation of ‘*urchin barrens’,* a rocky habitat dominated by crustose algae and encrusting invertebrates. Once formed, these urchin barrens can persist for decades. Trophic plasticity in the sea urchin may contribute to the stability and resilience of this alternate stable state by increasing diet breadth in sea urchins. This plasticity promotes ecological connectivity and weakens species interactions and so increases ecosystem stability. We test the hypothesis that sea urchins exhibit trophic plasticity using an approach that controls for other typically confounding environmental and genetic factors. To do this, we exposed a genetically homogenous population of sea urchins to two very different trophic environments over a period of two years. The sea urchins exhibited a wide degree of phenotypic trophic plasticity when exposed to contrasting trophic environments. The two populations developed differences in their gross morphology and the test microstructure. In addition, when challenged with unfamiliar prey, the response of each group was different. We show that sea urchins exhibit significant morphological and behavioural phenotypic plasticity independent of their environment or their nutritional status.

## Introduction

Grazing by regular sea urchins (Echinodea) plays a crucial role in structuring a range of marine habitats, and has been linked to phase shifts in tropical [1] and temperate [2] marine ecosystems. The change from kelp forest to rocky barrens is a vivid example of an ecosystem phase shift [3]. The phase shift is linked to changes in sea urchin populations: sea urchin barrens are associated with an increased sea urchin density [2], [4], [5] leading to increased grazing pressure, the formation of ‘grazing fronts’, removal of the kelp canopy and establishment of rocky barrens [6]. Their return to kelp forests is associated with reduced sea urchin densities [7]. The cause of such sea urchin population fluctuations are often a result of high levels of recruitment of juveniles to the habitat [8] or through the direct removal of sea urchin predators [9] or as a result of a trophic cascade [10].

Following a sea urchin induced phase shift, the trophic environment is radically altered in terms of the gross amount of food available and its nature. The trophic environment changes from a plentiful supply of easily accessible detached kelp fronds within the kelp forest, to one in which the sea urchins are scraping encrusting algae and invertebrates off rock [2], [11]. The populations of urchins within the new stability domain [12] are maintained either by the existing population or by successful juvenile recruitment to the locality following the phase shift [13], [14]. If the population persistence results from the maintenance of adult populations, then the adult sea urchins needs to exhibit a high degree of trophic plasticity to survive in the new stability domain. The longevity of sea urchin populations is poorly defined. However age estimates for some species are in excess of 100 years [15], and for *Psammechinus miliaris* used in this experiment is in excess of ten years [16], and so a single generation may easily persist for the duration of the phase shift.

Once a new stability domain is entered, the domain can persist for decades, with sea urchin populations remaining relatively stable following the initial boom [17], [18]. To maintain these barrens the sea urchins must display a high degree of trophic plasticity. Lawrence reported that of 201 sea urchin species, 103 (51%) species included animal and vegetable material in their diet [2]. Omnivory requires behavioural and morphological adaptation to feeding at multiple trophic levels [19], [20]: a component of this adaption will be genetic, and a component will be phenotypic. It has been shown that genetic variation can have a stabilising effect on food-webs [21]. In addition phenotypic plasticity can be a strong determinant of food chain structure [22]. This phenotypic variation of an individual in response to their trophic environment will act to widen its niche either through increasing individual diet breadth or by increasing the variation between individual diet breadth (sensu Roughgarden [23]). This increase in diet breadth promotes ecological connectivity and weakens species interactions [24]. Both of these traits have a strong stabilising effect on ecological networks [25], [26]. As such phenotypic trophic plasticity in sea urchins may be one mechanism that acts to maintain the urchin barrens once they form.

Sea urchins are well known for their morphological and behavioural plasticity. It has long been observed that populations from different habitats were morphologically distinct [27]. In addition, sea urchins have shown rapid morphological response to food availability [28], [29], substrate morphology [30], the presence of predators [31] and behavioural plasticity over environmental gradients [32]–[35]. Sea urchin barrens themselves are one such driver of morphological change, with populations of sea urchins of the same species having different morphologies inside and outside of sea urchin barrens [4]. In order to fully understand the role that phenotypic trophic plasticity in sea urchins plays in maintaining urchin barrens, it is first necessary to demonstrate that this plasticity is truly phenotypic. Secondly, we must show that the phenotypic plasticity is an adaption to the trophic environment unconfounded by other environmental factors, such as nutrient limitation or habitat differences. Thirdly, we need to understand if morphological adaption alters the way in which the sea urchins respond to their trophic environment in terms of prey handling and diet breadth. To do this we examined phenotypic plasticity in an echinoderm species: *Psammechinus miliaris.* This species is a small regular echinoid with a distribution along the north-eastern Atlantic from Scotland to North Africa. It is known to be strongly omnivorous [36], [37] and to exhibit a high degree of plasticity in the wild [38]. Under controlled laboratory conditions we challenged *P. miliaris* with two nutritionally equivalent, but physically different trophic environments and examined the nature of their phenotypic response, in terms of any changes in gross and microstructural characteristics and behaviour to their trophic environment.

## Methods

### Experimental Populations

The experimental populations of *Psammechinus miliaris* were spawned in the invertebrate hatchery at the Scottish Association for Marine Science. In April 2008, post metamorphosis (circa 1–3 mm diameter) the juvenile sea urchins were randomly separated into two populations and kept in four replicate aquaria. The sea urchins were fed one of two experimental diets. The ‘wild type’ diet consisted of fresh *Laminaria* spp. and whole mussels (*Mytilus edulis* 20–30 mm; shell length), reflecting the diet typically found in kelp beds The second diet was designed to be nutritionally equivalent, but to have very different material properties and require very different prey handling by the sea urchin to consume. The diet consisted of finely milled *Laminaria* spp. and mussel flesh in an agar binder (the processed diet). This diet has been previously used within the Scottish Association for Marine Science (SAMS) sea urchin production facility and was readily consumed by the sea urchins. Fresh *Laminaria spp*. and *M. edulis* were collected at approximately 2 week intervals from the Isle of Seil, west coast of Scotland and supplied *ad libitum* to the sea urchins. The ‘Prepared Diet’ was supplied to the sea urchins three times per week in excess of what could be consumed. The sea urchins were raised under these conditions until December 2009.

For comparison, wild sea urchins were collected from two locations in Loch Creran, which were approximately 2 km apart (Site 1 & 2) and each consisted of an intertidal and a subtidal population. Intertidal sea urchins were collected by hand at low tide on a boulder and sand habitat. Subtidal sea urchins were collected at each location from a depth of approximately 2 m and from a habitat of dense intact kelp, which runs parallel at a distance of ∼10 m to the intertidal population. Loch Creran is a Special Area of Conservation and not in private ownership, however no specific permits were required for the collections, and the species is not endangered or protected.

### Morphometric Analysis

Forty individuals from each of the four populations (2 experimental diets and 2 wild populations) were dissected to obtain morphometric data for allometric analysis. Nine linear and five mass characteristics were measured for each sea urchin. Linear characteristics included: test height, test diameter, diameter of peristomal opening and spine length (mean of five longest spines), test thickness (interambulacra) at ambitus (maximum test diameter) and lantern length and diameter were determined using digital vernier callipers to an accuracy of 0.01 mm. The measurement of the lantern length followed the procedure used by Black et al. (1982), where length was measured on freshly dissected, un-disarticulated lanterns from the shallow notch in the oral end of the pyramid to the slight depression in the aboral surface of the epiphysis. Mass characteristics included: wet mass (live), gonad wet mass, and lantern wet mass following 5 mins air-drying and were determined using laboratory scales to an accuracy of 0.01 g. Lantern dry mass was determined after 24 hrs drying in an oven at 105°C and lantern calcite free dry mass was determined by the method described in Hagen [39]. These characteristic were chosen as they have previously been used to describe morphological variation over a range of environmental parameters.

In addition, electron micrographs were used to examine the test plate microstructure. Ten sea urchins from each of the ‘wild diet’ and ‘processed diet’ populations were used. One interambulacral plate was removed from the ambitus of the eviscerated test, which had been dried to a constant weight at 60°C and cleaned in a 3 Mol solution of NaOH for 48 hours. The plates were rinsed with distilled water and allowed to dry prior to gold coating and examination under the SEM. The electron micrographs were then analysed using the ImageJ 1.44 (http://rsb.info.nih.gov/ij/) image analysis program. As plate microstructure is known to be heterogeneous across the face of the plate, each plate was divided into a central and peripheral region. From each region 30 non overlapping quadrats (100×100 µm) were randomly placed on the image and the number of pores per quadrat, the average pore area, and the pore circularity (circularity value of 1.0 indicates a perfect circle. As the value approaches 0.0 it indicates an increasingly elongated polygon) was calculated.

**Figure 1:**
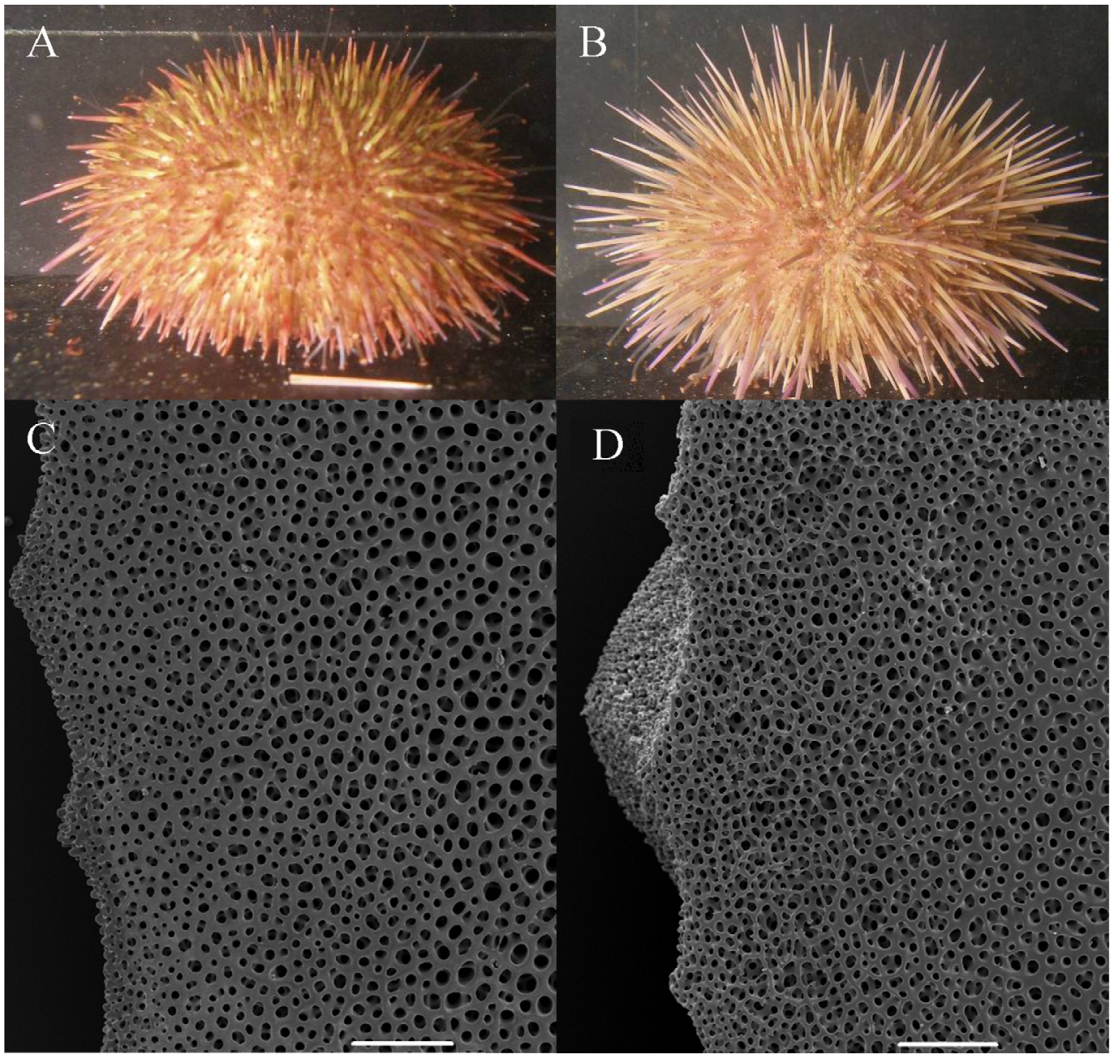
Gross morphological (panel A&B) and microstructural differences (panel C&D) in the test of *Psammechinus miliaris* from the wild type diets (A&C) and the processed diet (B&D). The sea urchins in panel A&B are approximately 40 mm test diameter. Scale bars on the SEM micrographs represent 200 µm.

All multivariate analysis was carried out using the PRIMER v6 (Plymouth Routines In Multivariate Ecological Research) statistical package. The data were normalised (the mean of the variable is subtracted from each data point and the resultant number is divided by the standard deviation of the variable) and converted into similarity matrices using Euclidean distances as the metric. Permutation based analysis of similarity (ANOSIM) routines were used as the hypothesis testing framework. Parametric analysis was conducted using GMAV5 (Institute of Marine Ecology, University of Sydney), all data was tested for homoscedasticity using Cochran’s test. Any data not meeting this assumption was transformed. Student Newman–Keuls post hoc testing was used where primary terms or interactions were significant at the 0.05 level. The responses in terms of time to successful predation were modelled using probit binary response and the binary logistic regression models in Minitab 14 was used to test for significant differences between the two treatments.

**Figure 2:**
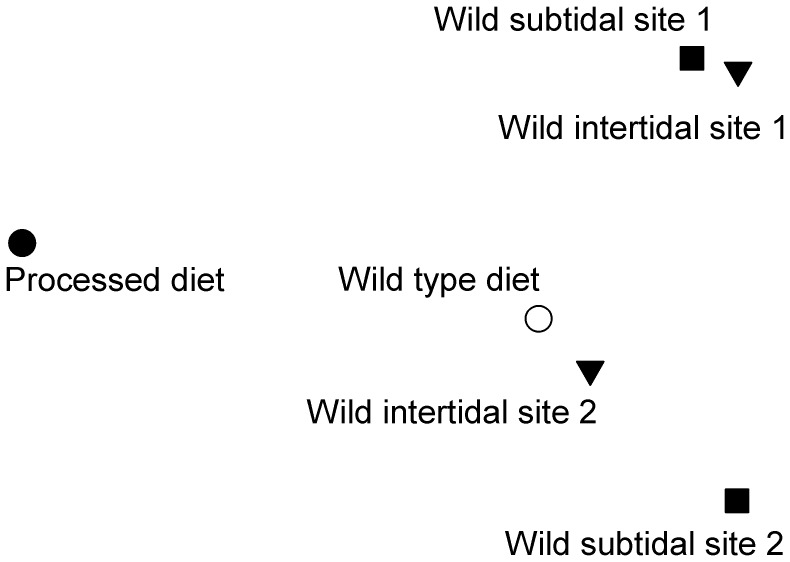
non Metric Multi-Dimensional Scaling plot of the morphometric data for all the populations of *P. miliaris,* using the multivariate dispersion R statistic from the paired *post-hoc* ANOSIM testing as the metric (○ wild type diet, •(black) processed diet, ▪(dark gray) subtidal site 1, ▾(dark gray) intertidal site 1, ▪ (light gray) subtidal site 2 ▾ (light gray)intertidal site 2).

### Behavioural Experiments

To test if the sea urchins from the two experimental treatments exhibited different prey handling behaviour a series of predator-prey interaction trials were conducted. Individual sea urchins from each of the ‘wild diet and ‘processed diet’ populations were placed in clear plastic aquariums with a single prey item. Then at regular intervals observations were made and note would be made as to whether a successful predation event had occurred. All prey items were bivalves and so a successful predation event was defined as the sea urchin having successfully opened the valves of the prey item. Three successive trials were conducted, and new animals were used in each trial; 1) small blue mussels (*Mytilus edulis*) (8–15 mm, n = 10 urchins from each treatment), 2) large blue mussels (25–35 mm, n = 20 urchins from each treatment), 3) pacific oyster (*Crassosttea gigas*) (20–30 mm n = 20 urchins from each treatment). The pacific oyster was used to expose the sea urchins to a novel prey species that neither population had prior experience.

**Table 1:** Multivariate analysis of the differences in morphology between the different populations of *P. miliaris*, based on post-hoc pairwise testing using a one way ANOSIM.

		Wild Type	Processed	Subtidal Site 1	Intertidal Site 1	Subtidal Site 2
Processed	R	0.287				
	p	0.001				
Sub tidal Site 1	R	0.192	0.506			
	p	0.001	0.001			
Intertidal Site 1	R	0.037	0.379	0.055		
	p	0.027	0.001	0.010		
Subtdal Site 2	R	0.184	0.402	0.068	0.058	
	p	0.001	0.001	0.002	0.007	
Intertidal Site 2	R	0.186	0.553	0.102	0.082	0.051
	p	0.001	0.001	0.002	0.002	0.008
		Wild Type	Processed	Subtidal Site 1	Intertidal Site 1	Subtidal Site 2

## Results

The sea urchins grew from post metamorphosis to an average test diameter 30.8 mm ± 1.7 mm (95% CI) for the wild type diet population and 32.6 mm ± 2.1 mm (95% CI) for the processed diets populations. There was no significant difference in the test diameter of the two populations (F_1,79_ = 1.77, p = 0.188). Mortality during this period was less than 5% for each population.

**Figure 3:**
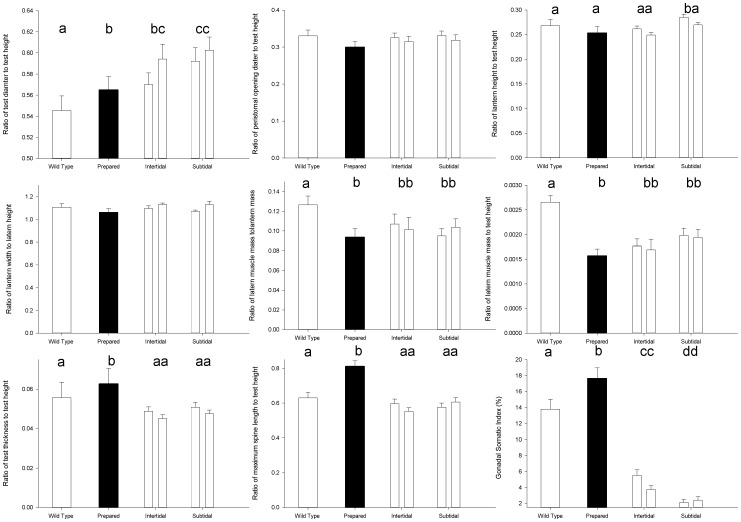
The morphometric comparison between all the different populations of *P. miliaris* (□ wild type diet, ▪ (black) processed diet, ▪ (dark gray) site 1, ▪ (light gray) site 2). The letters above indicate that significant differences exist between groups with different letters. Where there are no letters in the panel, there are no significant difference between any of the groups. Error bars represent 95% confidence intervals.

### Morphometrics

Sea urchins raised on the different diets showed marked differences in gross morphology and the microstructure of their tests (Figure 1). Multivariate statistics showed significant differences between the gross morphologies of all the sea urchin populations studied (Table 1). By examining the ANOSIM test statistic R, which is a measure of multivariate dispersion [40], it can be seen that, although still significantly different, the morphologies of the natural sea urchin populations more closely resemble the wild type diet treatment than processed diet treatment, when all the morphometrics are considered under a single multivariate analysis. Within the natural population, sea urchins from the two sites were more similar than those from the same habitats (Fig. 2, Table 1). There were also significant differences between the treatments and the natural populations in their univariate morphological characteristics (Fig. 3, Table 2).

**Table 2:** Analysis of variance of the differences in the microstructure of the tests between the two experimental populations of *P. miliaris.*

	Pores per unit area				Average pore size (×10^−3^)			Circularity		
Source	df	SS	F	p	SS	F	p	SS	F	p
Treatment	1	132.2	10.0	0.003	2.7	10.6	0.002	0.001	1.00	0.323
Position	2	59.6	2.3	0.114	0.03	0.03	0.971	0.021	7.00	0.002
Interaction	2	16.0	0.6	0.550	0.53	1.02	0.369	0.006	2.06	0.137
Error	54	712.1			24.23			0.079		
Total	59	919.8			27.44			0.107		

### Ratio of Test Height to Test Diameter

The test shape of the two populations was significantly (F_5,239_ = 11.0, p<0.001) different (Figure 3). For the sea urchins fed on the wild type diet the ratio of test diameter to height was significantly lower (describing a flatter shape) than for those urchins fed a processed diet, or from the intertidal of site 1. Those from the subtidal and the intertidal of site 2 had significantly rounder tests than the other populations.

**Figure 4:**
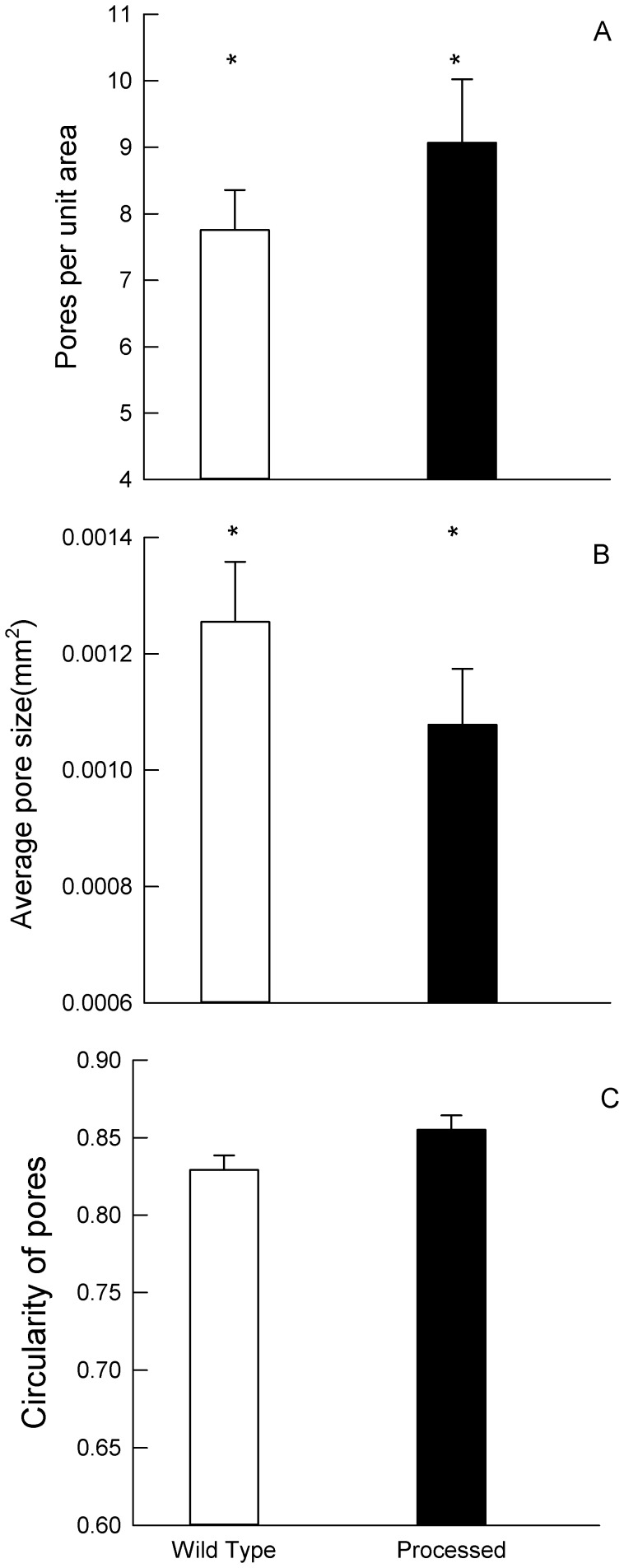
Differences in the microstructure of the tests of the two experimental populations of *P. miliaris* (□ wild type diet, ▪ (black) processed diet). Panel A) pores per unit area, B) average pore size mm^2^, C) pore circularity. * indicates a significant between the treatments.

### Ratio of Peristomal Opening to Test Height

There were significant differences amongst the populations (F_5,239_ = 2.76, p = 0.02). As post-hoc test was unable to discern amongst the groups, we can state that the processed diet population (the lowest) ratio was significantly different from the subtidal population from site 1 (the largest ratio).

**Table 3:** Binary logistic regression models for the differences prey handling response between the two treatments.

Small Mussel Trial				
Predictor	Coefficient	S.E. of Coefficient	Z	P
Constant	−0.892	0.202	−4.41	<0.001
Time	0.012	0.002	5.18	<0.001
Treatment	−0.350	0.253	−1.38	0.167
Test that all slopes are zero: G = 37.194, DF = 2, P-Value <0.001				
**Large Mussel Trial**				
Constant	−3.117	0.174	−17.93	<0.001
Time	0.003	0.004	7.30	<0.001
Treatment	0.854	0.135	6.31	<0.001
Test that all slopes are zero: G = 96.209, DF = 2, P-Value <0.001				
**Pacific Oyster Trial**				
Constant	−2.515	0.228	−11.04	<0.001
Time	0.007	0.001	6.49	<0.001
Treatment	−0.645	0.208	−3.10	<0.001
Test that all slopes are zero: G = 54.787, DF = 2, P-Value <0.001				

### Ratio of Lantern Height to Test Height

The subtidal population of site 1 had a significantly higher ratio of lantern height to test height (F_5,239_ = 9.93, p<0.001).

**Figure 5:**
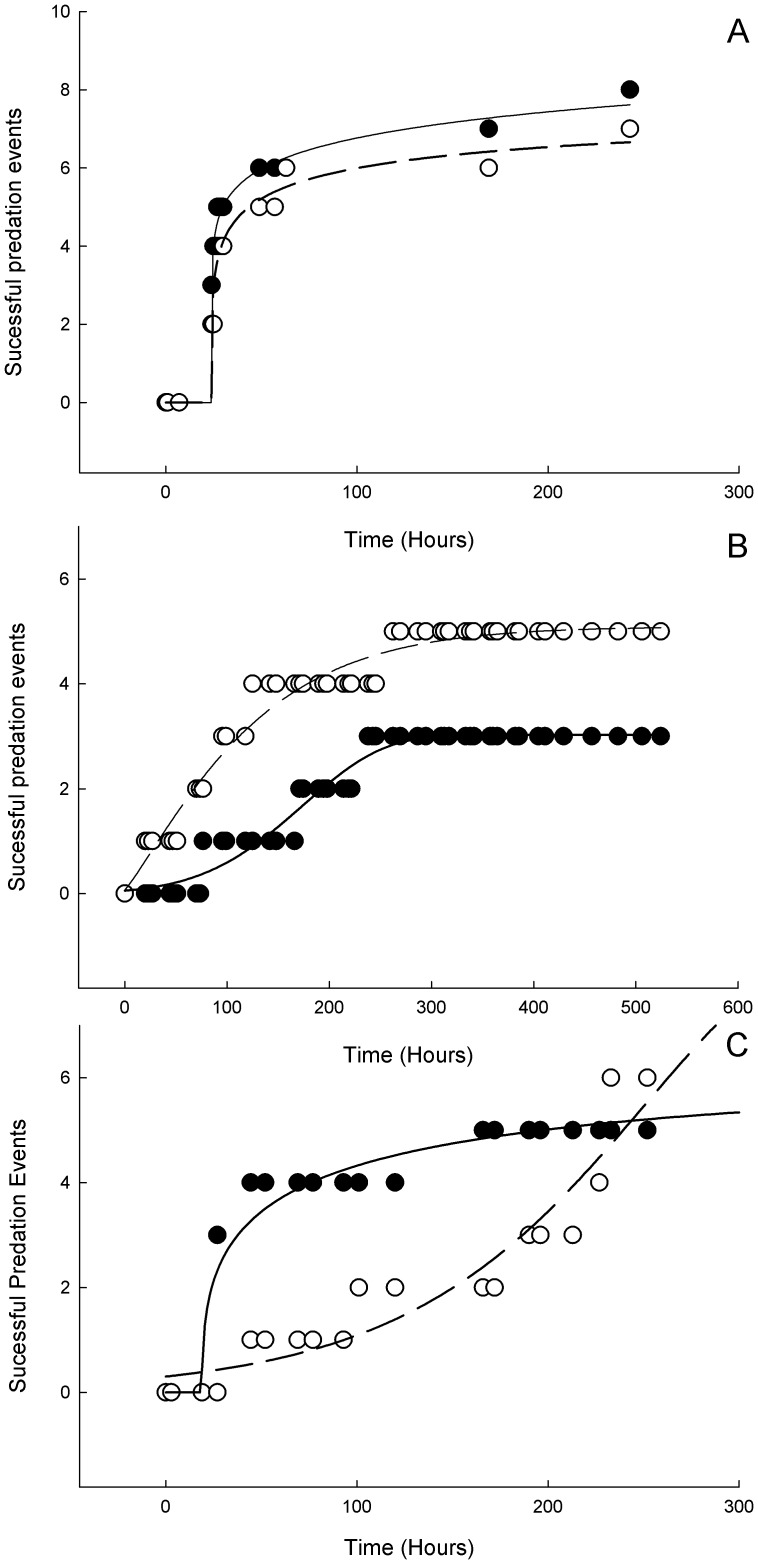
Probit binary regression models for the differences prey handling response between the two treatments for A) small *Mytilus edulis*, B) large *Mytilus edulis*, C) *Crassostrea gigas*. (○wild type, • (black) processed, circles represent real data points, fitted line from probit binary regression).

### Ratio of Lantern width to Lantern Height

There were significant differences between the population (F_5,239_ = 5.54, p = 0.001). As post hoc test was unable to discern amongst the groups, we can state that the processed diet population (the lowest) ratio was significantly different from the subtidal population from SS (the largest ratio).

### Ratio of Lantern Muscle Mass to Total Lantern Mass

The sea urchins fed on wild type diet had significantly higher ratio of lantern muscle mass to total lantern mass than any of the other populations (F_5,239_ = 5.88, p<0.001).

### Ratio of Lantern Muscle to Test Height

The sea urchins fed on wild type diet had significantly higher ratio of lantern muscle mass to total test height than any of the other populations (F_5,239_ = 14.73, p<0.001).

### Ratio of Test Thickness to Test Height

Relative to test height, sea urchins fed processed diets had thicker tests than any of the other populations (F_5,239_ = 8.91, p<0.001).

### Ratio of Maximum Spine Length to Test Height

Relative to test height, sea urchins fed processed diets had longer spines than any of the other populations (F_5,239_ = 43.62, p<0.001).

There were significant differences in the GSI between the wild type diet, the processed diet, the intertidal and the subtidal (F_5,239_ = 187.75, p<0.001). The sea urchins fed the processed diet had the highest GSI, followed by the wild type diet, the intertidal and then the subtidal.

### Microscopic Test Structure

Scanning electron micrographs of the test showed that the sea urchins fed on the wild type diet had significantly fewer and larger pores in their test compared to those urchins fed the processed diet. However the shape of the pores in terms of their circularity was not significantly different between the treatments (Table 2, Figure 4).

### Behavioural Response

There was no significant difference in the predator-prey response curves of sea urchin fed wild type diets and processed diet when presented with small mussels (Fig. 5A and table 3) with both species exhibiting a similar response curve. However when presented with large mussels (Fig 5B) and pacific oysters (Fig 5C), there was a significant difference in the prey handling response between the two experimental treatments. When confronted with large mussels, the wild diet type population had more successful predation events earlier in the observation period, compared to the processed diet population. The response to the non-native oyster (*Crassostrea gigas*) was more complex, the processed diet treatment initially opened more of the oysters at a faster rate, while the wild type population was slower to open them initially but by 300 hours they had more successful predation events.

## Discussion

When confronted with different trophic environments (diets that required very different prey handling attributes) but were nutritionally equivalent, a genetically homogenous population of sea urchins exhibited significant phenotypic plasticity in response. They developed gross morphological and microstructural differences to their tests and exhibited different prey handling behaviour. Such plasticity is well observed in sea urchins, but has been ascribed to a number of environmental or genetic drivers. Of the environmental drivers, nutrient limitation has received the greatest focus. Differences in the jaw structure [41]–[43], test thickness [43]–[45], and gonad size [29], [45], [46] have all been attributed to food limitation. In this current study phenotypic plastic adaptation has been shown in all these morphometric characteristics independent of nutrient limitation, showing that the phenotypic response of sea urchins to their trophic environment is more complex than previously thought.

Those sea urchins that were fed the processed diet developed morphologies distinct from those fed the wild type diet. This wild type diet morphology was closer to the morphologies of the natural populations as shown by the multivariate analysis, although several of the univariate morphometric measurements showed that there was no significant difference between the processed diet and the wild type diet populations, such as ratio of test diameter to test height, ratio of lantern muscle mass to total mass and the ratio of lantern muscle to test height. For these three morphometrics the response of the sea urchins fed on the wild type diet was greater than was observed in the population from the natural environment (i.e. they had flatter tests with more lantern muscle mass relative to either total mass, or test height). The diet of the natural populations of these sea urchins has been shown to consist of predominantly macroalgae and filter feeding invertebrates with intertidal populations consuming a higher proportion of filter feeding invertebrates compared to the subtidal [47]. This would suggest that the flattening of the test and the increase in lantern mass along with the other morphometric adaptions are a response to the physical requirements of handling the wild type diet. Phenotypic differences in morphologies of the sea urchin *Centrostephanus rodgersii* between kelp forests and urchin barrens have been previously reported [4], with those urchins from barren habitats having larger jaws, thinner tests, and longer spines. These differences in morphologies were attributed to nutrient limitation coupled with differences in the mechanical action of kelp fronds abrading spines between the habitats. However here we show that these differences can be generated solely as a result of differences in the prey handling characteristic of the diet. If a degree of the morphological plasticity observed by the experimental populations is in response to the nature of the diet (the principal differential between the two populations), then this variation can be seen as a development of a ‘tool kit’ required for handling and processing the wild diet of macroalgae and mussels. In this case the ‘tool-kit’ included a flatter test, increased lantern muscles and shorter spines. These attributes could all be seen as being advantageous when trying to open a bivalve. This is borne out in the prey handling experiments where sea urchins fed the wild type diet opened more, larger mussels, faster than those urchins fed processed diets (see below). In addition to the gross morphological characteristics, there were significant differences in the test microstructure between the experimental populations. Differences in test microstructure have been previously related to differences in growth rates as a result of differences in food availability [48]. However, this is the first record of differences in test microstructure independent of food availability. The sea urchins in the current study were of the same age and size, and there was no evidence that either population were nutrient limited, indeed both populations had GSIs far in excess of what was found in the natural populations. As the gonad is the primary nutrient store this suggests that neither population were nutrient limited and that variation in test micro-structure is independent of both growth rate and nutrition. This suggests that the altered structure is a phenotypic response to the two diet types.

There is also evidence that the sea urchins developed a behavioural plasticity according to their trophic environment. Sea urchins in the wild exhibit a range of behavioural plasticities in regards to their diurnal activity [49], covering behaviour [34] and their response to predators [50]. This however is the first record of plasticity in prey handling for regular echinoids. While there was no significant difference in the prey handling response for less challenging prey items for which the species had co-evolved (small blue mussels), when offered more challenging prey items of large blue mussels or Pacific oysters (which the *P. miliaris* had not coevolved with), there were significant differences in the prey handling response between the sea urchins fed the wild and the processed diets. These behavioural differences indicate that trophic plasticity in sea urchins is not just limited to a morphological response but they are also capable of adaptive behavioural responses.

The trophic environments that the two experimental populations were challenged with were very different in terms of the prey handling characteristics of the two diets. As a result the two populations exhibited a large degree of trophic plasticity in terms of their gross morphology, test microstructure and prey handling behaviour. We have shown that sea urchins are capable of exhibiting true phenotypic plasticity as a result of the trophic environment, independent of nutrient limitation. *P. miliaris* is known to be strongly omnivorous [36], [47], as are echinoids as a group. Therefore we propose that further work is required to test the hypothesis that phenotypic plasticity may act to increase the stability of an ecosystem and increase ecosystem resilience independent of total biodiversity, and that sea urchins with their exhibited morphological and behavioural trophic plasticity are ideal model organisms with which to test this hypothesis.
